# The Influence of the Home Food Environment on the Eating Behaviors, Family Meals, and Academic Achievement of Adolescents in Schools in the UAE

**DOI:** 10.3390/ijerph21091187

**Published:** 2024-09-06

**Authors:** Rahab Sohail, Hayder Hasan, Roba Saqan, Asmaa Barakji, Aisha Khan, Faaiza Sadiq, Shouq Furany, Zaina AlShaikh, Omar Atef Abdelhamid Mahmoud, Hadia Radwan

**Affiliations:** 1 Department of Clinical Nutrition and Dietetics, College of Health Science, University of Sharjah, Sharjah P.O. Box 27272, United Arab Emirates; rehabsohail99@gmail.com (R.S.); haidarah@sharjah.ac.ae (H.H.); asmaabarakji@hotmail.com (A.B.); aisha.khanus91@gmail.com (A.K.); fayizasadiq@gmail.com (F.S.); shoq.furany@hotmail.com (S.F.); zaina.alshaikh@hotmail.com (Z.A.); 2 Research Institute for Medical and Health Sciences, University of Sharjah, Sharjah P.O. Box 27272, United Arab Emirates; rsaqan@sharjah.ac.ae; 3 Lilehei Heart Institute, Cardiovascular Division, University of Minnesota, Minneapolis, MN 55455, USA; omahmoud@umn.edu

**Keywords:** adolescents, home food environment, eating behaviors, academic achievement, family meals, UAE

## Abstract

The eating behavior (EB) and habits developed during adolescence tend to persist into adulthood, with parents and caregivers playing a significant role in shaping their children’s food choices. The home environment is a crucial setting for developing eating behavior during adolescence. This study aimed to explore the influence of the home food environment (HFE) and its correlates on EB, family meals (FMs), and academic achievement among adolescents in schools in the United Arab Emirates (UAE). A cross-sectional study was conducted with 304 school-aged adolescents from the UAE. The questionnaire included sociodemographic data, dietary habits, information related to the HFE (food availability and accessibility), physical activity, sleep patterns, and academic achievement. Several questionnaire items were combined to create an HFE score. These questions included the frequency of weekly family meals, meal preparation practices, and accessibility to healthy and unhealthy food products and snacks at home. The HFE score was dichotomized into favorable and unfavorable HFE scores. Similarly, EB and FM scores were generated by combining responses to various related questions. The participants’ weights and heights were measured. The findings reported that more than half (55%) of the adolescents were either overweight or obese. The majority of the participants had favorable HFE (57.2%), EB (69.1%), and FM scores (58.2%). The significant correlates to the HFE were as follows: male participants whose parents attended college (OR: 0.31; 95% CI: 0.15–0.62; *p* < 0.001), high academic achievers (OR: 1.98; 95% CI: 1.02–3.82; *p* = 0.043), and those who were physically active (OR: 1.80; 95% CI: 1.14-2.85; *p* = 0.012), were more likely to have favorable HFE. Moreover, the HFE score showed a highly significant positive correlation with the EB score (r = 0.573, *p* < 0.001) and the FM score (r = 0.384, *p* < 0.001). These results underscore the critical role of a healthy HFE in shaping healthy positive eating behaviors and food choices among adolescents. They provide a foundation for developing effective, evidence-based policies that can impact the health and academic success of adolescents in the UAE.

## 1. Introduction

Adolescence is a critical period during which lifestyle behaviors and eating habits are shaped and maintained [1]. Unhealthy dietary habits and lifestyles among adolescents are considered risk factors for several nutrition-related diseases in adulthood [2]. Globally, teenagers frequently report low intake of fruits and vegetables, skipping meals, consuming considerable amounts of fast food in the diet, and not having meals with family [3]. Story and colleagues identified the food environment as a major component influencing adolescent eating behaviors [4]. Adolescent food environments are an important focus for interventions promoting healthy eating patterns. Specifically, the home food environment (HFE) serves as a primary source of calories and significantly impacts adolescents’ dietary habits [5]. Research reported that factors within the home food environment, such as the availability and accessibility of healthy and unhealthy foods at home, and the frequency and quality of family meals, contribute to the eating behaviors of children and adolescents [6,7]. Parents, often seen as the nutritional gatekeepers of the home, have a direct influence on the home food environment [8]. Parents and caregivers can impact their children’s dietary intake through role modeling and by shaping the food environment at home [9]. Findings from Project EAT (Eating and Activity over Time) suggest that family meals are associated with a better diet, fewer eating disorders, and improved psychosocial outcomes including academic achievement [10]. Adolescents who share family meals three or more times per week are more likely to have a healthy weight, follow a healthier diet, and are less prone to disordered eating [5,11]. Home food availability emerges as a determining factor in youth dietary outcomes [12]. While availability does not guarantee intake, limited availability of fruits and vegetables at home may hinder youths from meeting the dietary recommendations. Conversely, if high-calorie, tempting foods are readily available and accessible, their consumption may be more likely [5].

Research findings recognized optimal nutrition as a crucial factor affecting school performance and, consequently, achievements in the future [13]. Moreover, a few studies have examined the effects of diet quality on academic performance and reported that undernourished children have lower attendance, attention, and academic performance, as well as more health problems [14,15,16,17]. Furthermore, studies revealed that insufficient nutrient intake, particularly of iron, and excessive intake of fat and added sugars from fast food, are associated with poor academic performance and metabolic diseases, including insulin resistance and obesity [18]. Efforts to improve adolescent academic achievement are also being linked to frequent fruits and vegetable consumption, family meals, and regular physical activity [19].

It is noteworthy that most of the countries of the Eastern Mediterranean region (EMR), including the UAE, have undergone a rapid nutrition transition. This transition is characterized by the increased consumption of calorie-dense foods, fat (particularly animal fat), added sugars, and salt with decreased physical activity [20,21]. The economic growth in the UAE has contributed to significant changes in dietary habits and behavioral patterns among the citizens, which can be attributed to the rise of Western civilization [22]. The presence of numerous fast-food chains and the availability of easily accessible calorie-dense meals, among other factors, have contributed to the rising incidence of overweight, obesity, and noncommunicable diseases [22,23,24]. It was reported that less than one-third of Emirati adolescents met the recommendations for the intake of fruit and vegetables [25]. Further research emphasizes the importance of promoting dietary adequacy and variety, increased fruit and vegetable consumption, and moderate dietary fat intake as key nutrition messages for school-based programs [26]. Similarly, the HFE has been influenced by the rapid nutrition transition in countries of the Pacific Islands from traditional Pacific diets (starchy root crops, coconut, seafood, native fruits, and green leafy vegetables) toward diets high in processed foods [27].

Interventions to promote healthy eating habits among adolescents are particularly relevant in a country like the UAE given the rising rates of obesity and the related comorbidities among the youth population. The home food environment is a valuable area to explore when designing interventions aimed at improving adolescents’ diet quality. The identification of modifiable factors influencing adolescent eating behaviors is essential for designing effective interventions that promote healthy eating patterns in adolescents [28]. To develop targeted interventions, it is important to understand these behaviors within their socio-environmental context. There is a lack of research on how the home food environment influences the eating behaviors, family meals, and academic performance of adolescents in schools in the UAE. Therefore, the present study aimed to explore the influence of the home food environment (HFE) and its correlates on the eating behavior (EB), family meals (FMs), and academic achievement of adolescents in schools in the UAE. It is hypothesized that the home food environment significantly influences the eating behaviors, family meals, and academic achievement of adolescents in schools in the UAE.

## 2. Methods

### 2.1. Study Design

A quantitative cross-sectional study was carried out in public and private schools in Dubai and Sharjah, UAE, during the academic year of 2018–2019. A stratified cluster sampling was employed, with schools randomly selected based on their stratification (private vs. public). Four public and two private schools were selected in proportion to the overall ratio of private to public schools in the UAE. Clusters were defined as classrooms of grades 9–12.

### 2.2. Participants

The calculated sample size was 384 students assuming that the prevalence of the HFE is 50% (favorable vs. unfavorable), using a 5% marginal error and a level of statistical significance of 0.05 (two tailed). An additional 20% accounted for non-responses and control for the design effect, resulting a in sample size of 460. However, the total number of participants was 304, with a response rate of 66%.

The participants recruited in this study were students aged between 12 and 18 years old, attending grades (9–12) from the selected private and public schools in Dubai and Sharjah, UAE. The inclusion criteria were school students (boys and girls) aged between 12 and 18 years from different nationalities and whose parents agreed to and signed the consent form. The exclusion criteria included students outside this age group, those who did not complete the questionnaire or the anthropometric measurements, or whose parents did provide their consent.

This study was approved by the Research Ethics Committee (REC) of the University of Sharjah (reference number REC-19-05-13-01-S). Before recruitment, the school administration sent the informed consent form to the parents of the students of the selected classes, which included information about the study details, to be signed by the parents or guardians. Depending on the participants’ preferences and linguistic proficiency, the information sheet was distributed in either Arabic or English. During the school visit, the research assistants carefully read the information sheet to the consented students; explained the purpose of this study, its goals, and procedures in the classroom; and invited the students to participate. The students were then directed to the on-site school clinic, where the height and weight of each participant were measured.

### 2.3. Tools

A multicomponent questionnaire was completed by face-to-face interview by the research assistants during the school visit. The questionnaire was adapted from Project EAT (Eating and Activity over Time), which assesses the individual, environmental, and behavioral aspects associated with adolescents’ dietary intake and weight status [29]. Initially, the questionnaire was designed based on established frameworks and validated instruments used in similar studies. To tailor it to the UAE context, it was reviewed by a panel of experts, including nutritionists, public health professionals, and educators who have extensive experience working with adolescents in the UAE. Following the expert review, the questionnaire underwent a pilot test with a small group of adolescents from different cultural backgrounds within the UAE. This allowed us to assess the clarity, relevance, and cultural sensitivity of the questions. Feedback from the pilot test was incorporated to refine the questionnaire, ensuring that it was both culturally appropriate and understandable for the target population. This rigorous process helped to enhance the validity and reliability of the data collected in this study. The questionnaire was piloted on 30 students to confirm that it was culturally appropriate.

The questionnaire included questions related to sociodemographic information, HFE factors (home food availability and accessibility), family meals, eating habits, and physical activity. After that, the students were escorted to the school clinic to measure their weight and height.

#### 2.3.1. Sociodemographic

The sociodemographic section of the questionnaire collected data on age, sex, type of school, grade, parental educational attainment, and maternal employment.

#### 2.3.2. Home Food Environment (HFE)

The HFE was assessed based on questions related to home food availability, accessibility, and family meals. The HFE questions were derived and adapted from Project EAT [9,10]. The HFE score was developed by combining questions about the availability of and accessibility to healthy or unhealthy food items and snacks at home, the frequency of weekly family meals, and meal preparation practices. The questions included, for example, “I keep unhealthy snacks (potato chips, chocolate, candy, soda...) in my home”, “In my home, there is fresh fruit on the counter, table or somewhere where I can easily get it”, “In my home, there are cut-up vegetables in the fridge ready for me to eat”. The response options were yes or no. Meal preparation practices were assessed by asking: “Who prepares the meals at home?”. The response categories included mother, cook, helper, or others. The frequency of weekly family meals was assessed with the question: “During the past week, how many times did all, or most, of your family living in your house eat a meal together?”. The response options ranged from never to 1–3 times, 4–6 times, or every day. The HFE scores were categorized into binary outcomes, distinguishing between favorable and unfavorable HFE [29]. A value of (0) was assigned to indicate unfavorable HFE responses, whereas a value of (1) was assigned to indicate favorable HFE responses. Moreover, the variables indicating negative influences were reverse-coded so that a higher score would reflect a more favorable HFE. Then, the scores were totaled, and the cutoff point was determined using the median HFE score of 7.0 to dichotomize the total into unfavorable HFE (<7.0) and favorable HFE (≥7.0) [30].

#### 2.3.3. Eating Behaviors

The eating behavior data were assessed by asking participants about the frequency of meal consumption and specific foods in the past week. The questions included the following: “During the past week, how many days did you eat breakfast/lunch/dinner?’’ and ‘‘In the past week, how often did you eat something from a fast-food restaurant?”. The participants also reported the frequency of consumption of fruits and vegetables, as well as snacks (chips, chocolate, candy, and sugar-sweetened beverages). The response options were never, 1–3 days, 4–6 days, and every day. The details of the questions and responses are presented in Supplementary Table S1. An EB score was generated by combining the responses to these questions. A value of (0) indicated an unfavorable EB, while a value of (1) indicated favorable eating behavior. Then, the scores were totaled, and the cutoff point was determined using the median EB score of 6.0 to dichotomize the total into unfavorable EB (<6.0) and favorable EB (≥6.0) [30].

#### 2.3.4. Family Meals

The adolescents’ perception of family meals was assessed with questions about the priority, atmosphere, and structure of family meals as adapted from Project EAT [31]. The details of the questions and responses are presented in Supplementary Table S2. The responses were based on a 4-point Likert scale ranging from “strongly agree” to “strongly disagree”. An FM score was generated by combining the responses, with higher scores indicating more favorable FMs. A value of (0) indicated an unfavorable FM score whereas a value of (1) indicated a favorable FM score. Then, the scores were totaled, and the cutoff point was determined using the median FM score of 20.0 to dichotomize the total into unfavorable FM (<20.0) and favorable FM (≥20.0) [30].

#### 2.3.5. Lifestyle Factors

Physical activity was measured with the following question: “How many days do you exercise in a typical week?” (The responses varied from 0 to 7 days per week). The responses were then categorized (<2 times/week and ≥2 times/week). Also, parental influence on physical activity was evaluated with statements such as “My parents care about exercising and staying fit”. The responses included a 4-point Likert scale ranging from “strongly disagree” to “strongly agree” [32]. The purpose was to evaluate how parents influence their children’s physical activity and whether they encourage them or not.

Moreover, the participants reported their nightly sleeping duration, and the responses were categorized into <7 h and ≥7 h per day.

#### 2.3.6. Academic Achievement

The academic achievement of the participants was obtained from the school’s official records. The records included the student’s grades in all subjects from the previous year. Letter grades were converted into numeric values based on a 100% grading system (A = 90–100, B = 80–89, C = 70–79, D = 60–69, F < 60) and summed to generate an overall grade point average. The grades were categorized into two groups (<80% or ≥80%).

#### 2.3.7. Anthropometric Measurements

After completing the questionnaire, the participants were directed to the school clinic, where the school nurse measured their body weight (kg) and height (cm) using a Seca 220 Telescopic Measuring Rod. The measurements were taken twice for accuracy, with participants wearing clothing, without shoes, and with empty pockets. The body mass index (BMI) was calculated as weight in kg divided by height in meters squared (m^2^) and classified using the WHO growth charts: underweight BMI < 3rd percentile; normal weight: 3rd ≤ BMI ≤ 85th; overweight: 85th < BMI ≤ 97th; and obesity: BMI > 97th [33].

### 2.4. Data Analysis

Data analysis was performed using SPSS (Statistical Package for the Social Sciences) (Version 22, IBM Corp., Armonk, NY, USA). The descriptive statistics were presented as frequency and percentage for categorical variables and a median and interquartile range for continuous data. Simple and multiple logistic regression analyses were performed to identify the associations between factors and favorable HFE (compared to unfavorable HFE). The odds ratios (ORs) and their corresponding 95% confidence intervals (CIs) were calculated. For multiple logistic regression, only variables with *p*-values < 0.20 in the simple model were included in the final model. Further, Spearman correlation analysis was used to examine the correlation of HFE with EB and HFE with FM. A *p*-value of less than 0.05 was considered statistically significant.

## 3. Results

### 3.1. Descriptive Characteristics of the Schools’ Adolescents

Table 1 presents a summary of the characteristics of the adolescents. A total of 304 adolescents from public and private schools participated in this study with a median (IQR) age of 15.5 (2) years. More than half of the participants were girls (54%), attending public schools (59%) and their parents were college graduates (56%). The majority of the participants (63%) had high academic achievement (≥80%) and 51% reported sleeping for ≥7 h per day and exercising twice or more per week (51%). About 55% of the participants were either overweight or obese. The median (IQR) of the HFE, FM, and EB scores were 7.0 (2.0), 20.0 (5), and 6.0 (2), respectively, with the majority of the participants having favorable HFE (57.2%), EB (69.1%), and FM scores (58.2%).

### 3.2. Correlations of HFE Score with EB Score and Family Meal Score Using Spearman’s Rho Correlation

**Figure 1:**
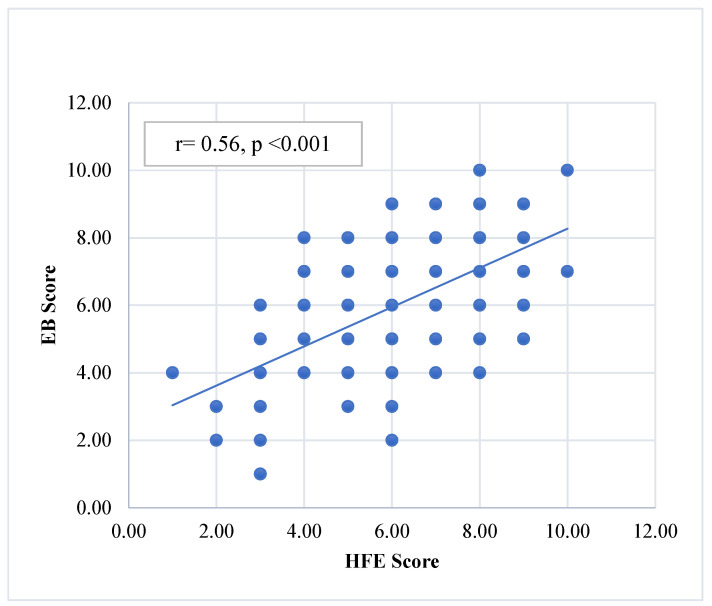
The correlation of the HFE score with the EB score.

**Figure 2:**
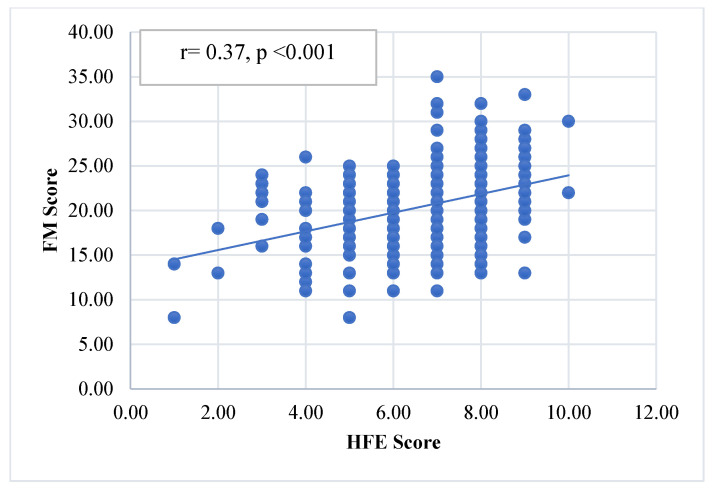
The correlation of the HFE score with the FM score.

Figure 1 and Figure 2 show the HFE correlations with the EB and FM scores. The HFE score showed highly significant positive correlations with both the EB and the FM scores [(r = 0.56, *p* < 0.001) and (r = 0.37, *p* < 0.001), respectively].

### 3.3. Determinants of the HFE (Favorable and Unfavorable) among the School Adolescents

Table 2 shows the simple and multiple logistic regression analysis to identify the determinants of the HFE. For the simple regression analysis results, boys who attended private school, were physically active, had high academic achievement, and whose parents were college graduates were more likely to have favorable HFE scores. The multiple logistic regression analysis revealed that the boys (OR: 0.31; 95% CI: 0.15–0.62; *p* < 0.001) who had high academic achievement (OR: 1.98; 95% CI: 1.02–3.82; *p* = 0.043) remained significantly associated with a favorable HFE. As for the EB and FM scores, both were highly significantly associated with the HFE (OR: 1.25; 95% CI: 1.15–1.36; *p* < 0.001) and (OR: 2.58; 95% CI: 2.00–3.33; *p* < 0.001), respectively.

## 4. Discussion

The present study reports novel findings on the home food environment in the UAE among adolescents in the UAE and its influence on their eating behavior, family meal patterns, and academic achievement. The cultural diversity and economic growth in the UAE have led to a dietary transition from traditional to more Western diets, which may have impacted the HFE, EB, and FMs of adolescents [21,34]. This research reveals significant associations between the HFE score and various factors, including sex, parents’ education level, academic performance, physical activity, FMs, and EB.

The findings of this study highlighted the high prevalence of overweight and obesity among school adolescents (55%). This prevalence is consistent with rates reported from other Arab countries ranging from 18% to 44% [35] and is lower than those in Western countries, where (22–25%) of European adolescents and 30% of American adolescents were either overweight or obese [36].

It was noted in this study that a large proportion of the adolescents whose parents attended college were more likely to have favorable HFE scores. This suggests that parental education influences the HFE in several ways including high earnings, financial planning, priority for nutrition, nutritional knowledge, parenting skills, and general assets [37]. Another research showed that the parents’ health practices, nutrition knowledge, and healthier food choices contributed to a healthier home [38]. Previous research has shown that the percentage of adolescents who eat a meal with their family seven or more times a week can be as low as 25% [39]. This finding implies the importance of parent education for family health besides the need to ensure that interventions centered on family meals are appropriate for and reach out to parents with lower levels of educational status [40]. Many adolescents do not eat with their families every day regardless of the many obvious benefits of family meals [39,41]. Corresponding to the results of one study involving 902 parents of adolescents, 79% of parents and 54% of adolescents reported having conflicting schedules that made it difficult to eat together [42]. Research has investigated potential obstacles perceived by both parents and adolescents, for instance, parents have emphasized schedule conflicts between employment and school [43].

In the current study, male participants were more likely to have a favorable HFE. This finding aligns with previous research indicating that boys often engage in healthier dietary behaviors and participate in family meals more frequently than girls. Earlier research reported that girls had less frequent family meals than boys [42]. In addition, a study found that males had healthier dietary habits than females [25]. For instance, a study on Indonesian adolescents found that males were more likely than females to choose healthier food options [44]. Additionally, research has shown that a significantly higher proportion of boys (59.3%) than girls (48.7%) was expected to eat all the foods served, even if they did not like them, suggesting stronger parental influence on boys’ eating habits [45]. A previous study in UAE, showed low rates of fruit and vegetable intake in Emirati adolescents, with a significantly lower intake frequency among girls [25]. Understanding these gender-specific dynamics is crucial for designing targeted interventions that address the unique needs of both male and female adolescents, ensuring that both groups benefit from a supportive home food environment.

Another finding of our study was that students attending private schools were more likely to have favorable HFE scores compared to those in public schools. This could be due to differences in the sociodemographic, environmental, and varied dietary patterns and the cultural diversity of the pupils attending private and public schools. In the UAE, the cultural differences and the diversity of students attending public and private schools may explain the variation in eating habits [24].

Studies conducted in Ethiopia and Brazil revealed that adolescents attending private schools consumed considerably more fruits and vegetables than their peers in public schools [46,47]. Addressing these differences is crucial for ensuring equitable access to healthy food environments across all school types.

Moreover, the findings of our study indicated that high academic achievers were more likely to have a favorable HFE score. This finding is supported by the existing literature that links the frequency of family meals with higher academic achievement [48]. A study reported that the frequency of family meals was inversely related to low grade point averages in adolescents [39]. Family meals might contribute to establishing bonding toward cultural and household practices, which can strengthen the cognitive health of adolescents [49]. It suggests that fostering a positive home food environment may contribute to better academic outcomes.

Regarding the relationship between exercise and HFE, this study indicated that participants who engaged in physical activity at least twice per week were more likely to have favorable HFE scores. These findings were consistent with a previous study that reported that adolescents living in a home environment offering high-fat snacks and spreads and fewer low-fat snacks and fruits and vegetables were associated with poor exercise and eating habits [50]. In another study, physical activity appeared to be associated with healthy choices, while sedentary behaviors were related more to unhealthy choices [51]. Moreover, higher consumption of fruits and vegetables was positively correlated with physical activity in other studies [52,53]. This association emphasizes the interconnectedness of physical activity and healthy eating habits, suggesting that interventions promoting both aspects could be more effective.

In the current study, the findings showed that a favorable HFE score was associated with a healthy family meal. Adolescents who ate with their families more frequently reported consuming healthier meals. Food availability at meals, mealtime rules, and the health-related attitudes of family members appeared to impact whether joining family meals would lead to a more nutritious diet [54]. This is probably because parents have the potential to impact their children’s eating and physical activity behaviors and thus reduce their risk for obesity through different practices such as supporting family meal frequency, purchasing different foods and serving them at meals, and other food socialization habits [55]. This highlights the role of family meals in establishing healthy eating habits and suggests that promoting regular family meals could be a key strategy in improving the home food environment.

The findings from this study underscore the critical role of the home food environment (HFE) in shaping healthy eating behaviors (EBs) among adolescents in the UAE. Adolescents’ access to a healthy HFE was most strongly and consistently associated with indicators of a healthy dietary intake [56]. According to a survey conducted in nearby countries such as Saudi Arabia, 55.7% of teens had unhealthy eating patterns (mostly fast-food ordering) up to three times per week [25]. Purchasing fast food for family meals was connected with various potentially unhealthy factors within the HFE and might be regarded as an indicator of a less ideal food environment in the home [55]. Correspondingly, another research reported that the availability and accessibility of fruits and vegetables at home led to an increase in their consumption [57]. Furthermore, a recent review revealed that the frequency of family meals was found to be inversely related to the consumption of fast food and unhealthy snacks and was associated with increased consumption of fruits and vegetables [40]. Many studies have reported that unhealthy eating behaviors among adolescents could be associated with the development of chronic non-communicable diseases during adulthood, if not corrected [58,59,60]. The strong association between a favorable HFE and indicators of healthy eating behaviors suggests that the home food environment may serve as a protective factor against unhealthy eating behaviors and influence their long-term health and well-being.

While this study provides valuable insights, several limitations should be acknowledged. The small sample size may limit the generalizability of the findings to the broader adolescent population in the UAE. Future studies should aim to include larger, more representative samples to validate these findings. Moreover, the cross-sectional nature of the current study restricts the ability to establish causality. Longitudinal studies are needed to better understand the directionality of the relationships between HFE, EB, and other factors such as family meal patterns and academic achievement. Further, relying on self-reported information related to the HFE, EB, and FMs, this study can be susceptible to both social desirability bias and recall bias. Moreover, another limitation that was not fully explored was the parental influence. Although this study acknowledges the role of parents as nutritional gatekeepers, it did not delve deeply into the specific parental practices or attitudes that may influence the home food environment and adolescent eating behaviors potentially offering more nuanced insights into how parental behaviors shape adolescent eating habits.

## 5. Conclusions

This study’s findings highlighted the significant role of a healthy eating environment at home in promoting healthy behaviors among adolescents. Fostering a favorable HFE will positively influence eating behaviors, family meal patterns, and academic achievement. The home food environment is a valuable area to explore when attempting to target interventions for improving the youth population. Nutritionists and public health educators should focus on promoting the importance of the HFE in influencing various aspects of adolescents’ lives, including their dietary habits and academic performance. Those studies should focus on parent diet quality and knowledge and how that could potentially affect the observed relationship between the HFE and healthy family meal patterns. The views of both adolescents and parents are essential for the effective implementation of family-oriented nutrition promotion strategies. School-based programs should be designed to involve both adolescents and their parents, to raise awareness and improve their food and nutrition literacy about the importance of creating healthier food environments at home. Future research should aim to clarify the directionality of the associations observed in this study through longitudinal designs and explore the impact of parental diet quality and knowledge on HFE. By targeting the HFE and related factors, such as family meals and parental influence, public health initiatives can make significant strides in improving the dietary habits and overall health of adolescents in the UAE.

## Figures and Tables

**Table 1:** Sociodemographic characteristics, lifestyle factors, BMI, and eating attitude scores of the participants (n = 304).

Characteristics	N (%)
Age	15.50 (2) *
Sex	
Boys	140 (46)
Girls	164 (54)
School Type	
Public	179 (59)
Private	125 (41)
Grade	
7–8	21 (7)
9–10	144 (47)
11–12	139 (46)
Mother’s Education	
No school	10 (3)
School level	117 (39)
College level	177 (58)
Father’s Education	
No school	18 (6)
School level	107 (35)
College level	179 (59)
Mother’s Occupation	
Unemployed	192 (63)
Employed	112 (37)
Academic Performance	
<80%	111 (37)
≥80%	193 (63)
Physical Activity	
<2 times/week	150 (49)
≥2 times/week	154 (51)
Sleep	
<7 h/day	150 (49)
≥7 h/day	154 (51)
BMI	
Underweight	5 (2)
Normal	131 (43)
Obese/overweight	168 (55)
Home Food Environment Score	7.0 (2.0) *
Favorable HFE score (≥7.0)	174 (57.2)
Unfavorable HFE score (<7.0)	130 (42.8)
Family Meal Score	20.0 (5.0) *
Favorable FM score (≥20.0)	177 (58.2)
Unfavorable FM score (<20.0)	127 (41.8)
Eating Behavior Score	6.0 (2.0) *
Favorable EB score (≥6.0)	210 (69.1)
Unfavorable EB score (<6.0)	94 (30.9)

* Median ± IQR. Abbreviations: IQR: interquartile range; EB: eating behavior, FM: family meal, HFE: home food environment, BMI: body mass index.

**Table 2:** The determinants of the HFE among 304 participants.

	Simple Logistic Regression		Multiple Logistic Regression ^a^	
	OR (95% CI)	*p*-Value	OR (95% CI)	*p*-Value
Age	1.05 (0.88, 1.25)	0.581		
Sex Ref [Boys]				
Girls	0.52 (0.33, 0.83)	0.006	0.31 (0.15, 0.62)	<0.001
School Type Ref [Public]				
Private	1.80 (1.13, 2.89)	0.014	0.55 (0.23, 1.33)	0.186
Grade Ref [Grades:7,8]				
9–10	0.91 (0.36, 2.29)	0.844		
11–12	1.11 (0.44, 2.81)	0.823		
Mother’s Education Ref [No school]				
School level	4.36 (0.89, 21.39)	0.070	4.03 (0.41, 39.93)	0.233
College level	6.73 (1.39, 32.63)	0.018	6.85 (0.66, 70.50)	0.106
Father’s Education Ref [No school]				
School level	2.85 (0.95, 8.57)	0.061	2.67 (0.54, 13.28)	0.231
College level	4.45 (1.52, 13.05)	0.006	3.12 (0.60, 16.19)	0.176
Mother’s Occupation Ref [Unemployed]				
Employed	0.79 (0.49, 1.26)	0.324		
Academic Performance Ref [<80%]				
≥80%	1.64 (1.02, 2.63)	0.041	1.98 (1.02, 3.82)	0.043
Physical Activity Ref [<2 times/week]				
≥2 times/week	1.80 (1.14, 2.85)	0.012	0.89 (0.47, 1.69)	0.728
Sleep Ref [<7 h]				
≥7 h	0.80 (0.51, 1.26)	0.337		
BMI Ref [Underweight]				
Normal	1.95 (0.31, 12.05)	0.473		
Obese/overweight	2.10 (0.34, 12.90)	0.423		
FM Score	1.23 (1.15, 1.32)	<0.001	1.25 (1.15, 1.36)	<0.001
EB Score	2.24 (1.82, 2.75)	<0.001	2.58 (2.00, 3.33)	<0.001

Abbreviations: Ref: reference, OR: odds ratio, CI: confidence interval, HFE: home food environment, BMI: body mass index. ^a^ Variables with a *p*-value less than 0.2 in the simple regression analysis were included in this regression.

## Data Availability

The data supporting this study’s findings are available upon request from the corresponding author. The data cannot be made publicly available due to privacy and ethical considerations.

## Supplementary Materials

The following supporting information can be downloaded at https://www.mdpi.com/article/10.3390/ijerph21091187/s1, Table S1. Eating behaviors of the schools’ adolescents in the UAE; Table S2. Adolescents’ perception of family meal patterns.
