# Supplementary material for: The Influence of the Home Food Environment on the Eating Behaviors, Family Meals, and Academic Achievement of Adolescents in Schools in the UAE
Source: Int J Environ Res Public Health. 2024 Sep 6;21(9):1187. doi: 10.3390/ijerph21091187 (PMC11431298; doi:10.3390/ijerph21091187)
Supplement: Supplementary file 1 [file ijerph-21-01187-s001.zip › ijerph-3139410-supplementary.pdf]

Supplementary Materials

Table S1. Eating behaviors of the schools' adolescents in the UAE.

Participants' Eating Behaviors	Never	1–3 Times	4–6 Times	Everyday
Breakfast frequency/week	62 (20.4%)	104 (34.2%)	30 (9.9%)	108 (35.5%)
Lunch frequency/week	5 (1.6%)	56 (18.4%)	50 (16.4%)	193 (63.5%)
Dinner frequency/week	16 (5.3%)	78 (25.7%)	61 (20.1%)	149 (49%)
Snack frequency/day	75 (24.7%)	144 (47.4%)	42 (13.8%)	43 (14.1%)
App. usage for ordering/week	120 (39.5%)	147 (48.4%)	25 (8.2%)	12 (3.9%)
Fast-food intake/week	120 (39.5%)	159 (52.3%)	18 (5.9%)	7 (2.3%)
Sugary drink frequency/week	78 (25.7%)	140 (46.1%)	44 (14.5%)	42 (13.8%)

Table S2. Adolescents' perception of family meal patterns.

Family Meal Patterns	Strongly Disagree	Disagree	Agree	Strongly Agree
Parents care about healthy eating	5 (1.6%)	43 (14.1%)	174 (57.2%)	82 (27%)
Parents care about exercise	8 (2.6%)	68 (22.4%)	158 (52%)	70 (23%)
Importance of family mealtime at least once a day	23 (7.6%)	63 (20.7%)	116 (38.2%)	102 (33.6%)
Talking during mealtime	16 (5.3%)	64 (21.1%)	145 (47.7%)	79 (26%)
Watching TV during mealtime	79 (26%)	117 (38.5%)	80 (26.3%)	28 (9.2%)
Expectation to be home for dinner	45 (14.8%)	125 (41.1%)	94 (30.9%)	40 (13.2%)
Different schedules a barrier to family meals	57 (18.8%)	101 (33.2%)	96 (31.6%)	50 (16.4%)
Eating together is enjoyable	25 (8.2%)	52 (17.1%)	149 (49%)	78 (25.7%)
Finishing plate despite dislike	66 (21.7%)	116 (38.2%)	75 (24.7%)	47 (15.5%)
Eating at a table is not mandatory	50 (16.4%)	70 (23%)	140 (46.1%)	44 (14.5%)
Eating something other than the food served	42 (13.8%)	79 (26%)	137 (45.1%)	46 (15.1%)
Healthy foods do not taste good	67 (22%)	95 (31.3%)	95 (31.3%)	47 (15.5%)
